# Impaired expression of serine/arginine protein kinase 2 (SRPK2) affects melanoma progression

**DOI:** 10.3389/fgene.2022.979735

**Published:** 2022-09-23

**Authors:** Mônica Maria Magalhães Caetano, Gabriela Alves Moreira, Maria Roméria da Silva, Gabriela Rapozo Guimarães, Leandro de Oliveira Santos, Amanda de Ambrósio Pacheco, Raoni Pais Siqueira, Flávia Carneiro Mendes, Eduardo De Almeida Marques Da Silva, Abelardo Silva Junior, Juliana Lopes Rangel Fietto, Ângela Saito, Mariana Boroni, Gustavo Costa Bressan

**Affiliations:** ^1^ Departamento de Bioquímica e Biologia Molecular, Universidade Federal de Viçosa (UFV), Viçosa, Brazil; ^2^ Laboratório de Bioinformática e Biologia Computacional, Divisão de Pesquisa Experimental e Translacional, Instituto Nacional de Câncer (INCA), Rio de Janeiro, Brazil; ^3^ Departamento de Biologia Geral, Universidade Federal de Viçosa (UFV), Viçosa, Brazil; ^4^ Departamento de Veterinária, Universidade Federal de Viçosa (UFV), Viçosa, Brazil; ^5^ Laboratório Nacional de Biociências (LNBio), Centro Nacional de Pesquisa em Energia e Materiais (CNPEM), Campinas, Brazil

**Keywords:** melanoma, metastasis, cancer, SRPK1, SRPK2, actin, B16F10

## Abstract

Melanoma is one of the most aggressive tumors, and its lethality is associated with the ability of malignant cells to migrate and invade surrounding tissues to colonize distant organs and to generate widespread metastasis. The serine/arginine protein kinases 1 and 2 (SRPK1 and SRPK2) are classically related to the control of pre-mRNA splicing through SR protein phosphorylation and have been found overexpressed in many types of cancer, including melanoma. Previously, we have demonstrated that the pharmacological inhibition of SRPKs impairs pulmonary colonization of metastatic melanoma in mice. As the used compounds could target at least both SRPK1 and SRPK2, here we sought to obtain additional clues regarding the involvement of these paralogs in melanoma progression. We analyzed single-cell RNA sequencing data of melanoma patient cohorts and found that SRPK2 expression in melanoma cells is associated with poor prognosis. Consistently, CRISPR-Cas9 genome targeting of SRPK2, but not SRPK1, impaired actin polymerization dynamics as well as the proliferative and invasive capacity of B16F10 cells *in vitro*. In further *in vivo* experiments, genetic targeting of SRPK2, but not SRPK1, reduced tumor progression in both subcutaneous and caudal vein melanoma induction models. Taken together, these findings suggest different functional roles for SRPK1/2 in metastatic melanoma and highlight the relevance of pursuing selective pharmacological inhibitors of SRPK2.

## 1 Introduction

Melanoma is associated with aggressive histopathologic features and poor prognosis due to its high metastatic capacity and the limited therapeutic tools available to prevent the disease progression. Indeed, melanoma is considered the deadliest type of skin cancer (Guo et al., 2021). Abnormal activity of SRPKs, mostly SRPK1 and SRPK2, has been found in different cancer types (Gammons et al., 2014; Siqueira et al., 2015; van Roosmalen et al., 2015; Bullock et al., 2016; Gong et al., 2016; Sigala et al., 2016; Zhuo et al., 2018), where they act in cellular processes related to tumor development and metastasis, such as angiogenesis (Gammons et al., 2014), epithelial-mesenchymal transition (EMT) (Wang et al., 2017), and cell migration (van Roosmalen et al., 2015; Wang et al., 2016). In melanoma cell lines, high SRPK1 expression favors angiogenesis through splicing regulation of a pro-angiogenic vascular endothelial growth factor (VEGF) isoform (Gammons et al., 2013).

SRPK1 and SRPK2 respond to epidermal growth factor receptor (EGFR) activity in the context of the phosphoinositide 3-kinase (PI3K)-AKT-SRPK signaling axis, connecting the extracellular EGF signal to the nucleus through the phosphorylation of the SR family of splicing factors (Zhong et al., 2009; Zhou et al., 2012). Despite this observation, SRPK1 and SRPK2 seem to act in different ways in cancer, depending on the cell type in which they are expressed. High levels of SRPK1 have been correlated with metastasis formation and poor prognosis in breast cancer (van Roosmalen et al., 2015). In colorectal cancer, SRPK1 mediates the nuclear import of SRSF1, which regulates tumor-related alternative splicing of Rac1b, a small, constitutively active GTPase with pro-tumor functions (Gonçalves et al., 2014; Gudiño et al., 2021). On the other hand, the effect of SRPK2 on colon cancer cell growth and migration has been shown to be mediated by the extracellular-related kinase (ERK) signaling pathway (Wang et al., 2016). SRPK2 has been revealed as the effector kinase involved in mammalian target of rapamycin complex 1 (mTORC1)-dependent regulation of lipid metabolism, an important process that supports cancer cell proliferation (Lee et al., 2017).

The wide action of SRPK1/2 in different stages of tumorigenesis suggests they are promising antitumor therapeutic targets (Gammons et al., 2013; Morooka et al., 2015; Siqueira et al., 2015; Batson et al., 2017; Hatcher et al., 2018). SRPIN340 was the first discovered SRPK inhibitor with antiviral potential (Fukuhara et al., 2006). In the tumor context, we have demonstrated that SRPK1/2 pharmacological inhibition by the classical inhibitor SRPIN340 and other derivatives decreased the invasive capacity of metastatic melanoma B16F10 cells *in vitro* and reduced the formation of pulmonary nodules *in vivo*, which can be related to immune system activation (Moreira et al., 2018; Mendes et al., 2022; Moreira et al., 2022). However, as SRPIN340 inhibits SRPK1 and to a lesser extent SRPK2, as well other splicing kinases (Fukuhara et al., 2006), these data are limited in informing which SRPK1/2 member is relevant for melanoma progression. Therefore, we designed the present study to evaluate whether these important splicing kinases are involved differentially in melanoma development.

## 2 Materials and methods

### 2.1 Single-cell analysis

We downloaded clinical and pre-processed single-cell RNA sequencing (scRNA-Seq) public data (Jerby-Arnon et al., 2018) of patients with melanoma and processed it according to the scripts available in the GitHub repository “SRPK at single-cell resolution” (Rapozo and Santos, 2022a). We used the previous annotation of the Seurat object (Butler et al., 2018) to identify all the cells of the tumor microenvironment (TME) (Moreira et al., 2022). Melanoma is largely related to DNA copy number changes (Shain et al., 2015). Hence, we distinguished malignant cells from normal melanocytes using inferCNVpy (https://github.com/icbi-lab/infercnvpy) in order to generate single-cell copy number variant (CNV) profiles. These cells were classified as “High_CNV” or “Low_CNV” according to the cnv signal, using a cutoff of 0.006. We determined this cutoff by calculating the average score of all cell types in the TME; we classified the cells with a percentage greater than this value as malignant.

### 2.2 Digital cytometry and survival analysis

To study the clinical impact of malignant cells expressing SRPK, we downloaded a bulk RNA-Seq expression, and clinical-pathological data from skin cutaneous melanoma (SKCM) cohort from The Cancer Genome Atlas (TCGA) database http://cancergenome.nih.gov/ using the TCGA biolinks package in the R environment (Colaprico et al., 2016). Next, we estimated the proportions of cell subtypes found in single-cell analysis (broad cell types and malignant High_SRPK and Low_SRPK) in these bulk RNA-Seq samples through a deconvolution approach using a digital cytometry tool, the BisqueRNA (Jew et al., 2020). Next, to evaluate the impact of all cell types on survival, we calculated the hazard ratio (HR) of the relative fractions of each cell type population divided into quartiles, with 95% confidence intervals (CIs) based on maximum likelihood estimates for each covariate using a univariate Cox regression model (Cox, 1972) (*p* < 0.02) to find the significant variables to be tested in the multivariate Cox regression model (*p* < 0.05). For these analyses, we used the Survival and Survminer packages for the R environment (Kassambara et al., 2017; Therneau, 2022).

### 2.3 Antibodies, plasmid, and cell culture

We used the following antibodies in this study: mouse monoclonal anti-SRPK1 and anti-SRPK2 (BD Biosciences), mouse monoclonal anti-GAPDH (Invitrogen), rabbit anti-Ki67 (Abcam), Alexa Fluor 488–conjugated goat anti-rabbit IgG (Thermo Fisher Scientific), and horseradish peroxidase (HRP)–conjugated secondary antibodies (Sigma). We obtained LentiCRISPRv2 from Addgene (#52961) (Sanjana et al., 2014). Mouse melanoma B16F10 cells were kindly provided by Dra. Anésia Aparecida dos Santos (Departamento de Biologia Geral, Universidade Federal de Viçosa, Minas Gerais, Brazil). We cultured cells in RPMI-1640 supplemented with 10% fetal bovine serum (FBS, LGC Biotecnologia) and 3 mM L-glutamine (Sigma), 100 U/mL penicillin, and 100 μg/mL streptomycin (Sigma), at pH 7.2 and 37°C under a 5% CO_2_ atmosphere.

### 2.4 Genome-editing procedures

We used clustered regularly interspaced short palindromic repeat (CRISPR) genome editing to target SRPK1, SRPK2, or both, in B16F10 cells (B16F10 SRPK^−^). We designed each single guide RNA sequence (sgRNA) targeting murine SRPK1 (TTA​CCG​GTC​TCG​CCA​TGG​AG) or murine SRPK2 (AGG​CTG​TCT​CTG​TAT​AAT​GC) using the online CRISPR design tool at http://crispr.mit.edu (Hsu et al., 2013). We cloned complementary oligo sgRNAs into the BsmBI restriction site of LentiCRISPRv2 that we used for lentivirus particle production. We transduced the lentiviral vector into target cells with a multiplicity of infection (MOI) of 1 in the presence of 10 µg/mL polybrene (Sigma). We used LentiCRISPRv2 without sgRNA as an empty vector for mock transduction (named Control). The media was changed 24 h post-transduction to select transduced B16F10 cells with 2 µg/mL puromycin (Sigma). Every 2 days, the medium was replaced with RPMI complete with puromycin to eliminate the puromycin-sensitive cells. To simultaneously target SRPK1 and SRPK2, we transduced the already obtained B16F10 SRPK2^-^ targeted cells to target SRPK1. The cells were allowed to grow for 1 week prior to expression analysis by western blotting.

### 2.5 Colony formation assay

We seeded B16F10 modified cells in 6-well plates in triplicate at the density of 1.0 × 10^3^ cells per well. The cells were allowed to grow in RPMI-1640 medium supplemented with 2% fetal bovine serum for 14 days. We then fixed colonies, stained them with toluidine blue solution (0.5% v/v) and methanol (20% v/v), and counted them.

### 2.6 Transwell invasion assay

We coated transwell inserts with 8-μm pores (Corning Life Sciences) with Matrigel (BD Bioscience). Then, 5 × 10^4^ B16F10 modified cells diluted in 200 µl of serum-free medium were seeded in the upper chambers. The lower chamber was filled with 650 μl of culture medium containing 10% v/v FBS as a chemoattractant. After 24 h, we fixed, washed, and stained the chambers as described previously (Moreira et al., 2018). We counted the cells using an inverted microscope (Leica).

### 2.7 Cell staining and fluorescence microscopy

We seeded cells on 24-well plates containing coverslips and treated them with EGF 100 ng/mL (Sigma) for 1 h when indicated (Zhou et al., 2012). After 24 h, we fixed, permeabilized, and blocked the cells as published previously (Moreira et al., 2018). For indirect fluorescence staining of Ki67, we incubated cells with anti-Ki67 antibody for 1 h. After washing with phosphate-buffered saline (PBS), we incubated the cells with Alexa Fluor 488-conjugated goat anti-rabbit IgG for 45 min and then washed again with PBS. To visualize actin filaments, we stained the cells with rhodamine-phalloidin (Thermo Fisher Scientific) for 20 min. After incubation, we washed the cells with PBS and mounted the coverslips on microscope slides using Prolong Diamond Antifade Reagent with DAPI (Invitrogen). We examined the fluorescence using an inverted fluorescence microscope (EVOS FL).

### 2.8 Western blotting

We prepared cell lysates in lysis buffer containing 20 mM Tris (pH 7.5), 1% (v/v) NP40, 5 mM EDTA, protease and phosphatase inhibitors (Sigma), and 150 mM NaCl, as described in our previous work (Siqueira et al., 2015). The proteins were resolved by 12% sodium dodecyl sulfate (SDS)–polyacrylamide gel electrophoresis and transferred to a nitrocellulose membrane (GE Healthcare). The blots were incubated with 3% bovine serum albumin (BSA) in 1X TBST buffer (1X Tris-Buffered Saline, 0.1% Tween 20) and then incubated with primary antibodies. The blots were washed three times with TBST buffer and incubated with secondary antibodies. We visualized the proteins using Amersham ECL Prime Western blotting detection reagent (GE Healthcare), under a digital scanner C-DiGit Blot (LI-COR). We performed densitometry analysis of the band intensity using ImageJ software.

### 2.9 *In vivo* subcutaneous tumor and metastasis models

We obtained male C57BL/6 mice from the Central Animal Laboratory (Centro de Ciências Biológicas e da Saúde, Universidade Federal de Viçosa, Minas Gerais, Brazil) and maintained them under controlled temperature (21.2°C), relative humidity of 60%–70%, and a 12-h photoperiod. The animals received food and water *ad libitum*. The experimental protocols were approved by the Ethics Committee of Animal Use of the Universidade Federal de Viçosa (CEUA/UFV, protocol 30/2019). For analysis of subcutaneous tumor growth, we subcutaneously inoculated B16F10 modified cells into the right flank of mice (*n* = 10 animals/group) (Vale et al., 2020). We measured the tumor volume (V) three times a week using formula V = 0.52 × D1 × D2, where D1 and D2 are the short and long tumor diameters, respectively (Figueiredo et al., 2015). On day 15, we euthanized the mice by deep anesthesia (intraperitoneal injection of ketamine hydrochloride 150 mg/kg body weight and xylazine hydrochloride 10 mg/kg body weight), followed by cardiac puncture.

For the induction of lung metastasis, we inoculated B16F10 modified cells into the tail vein of the mice (n = 10 animals/group). All procedures, including euthanasia, lung collection, and nodule count, have been described previously (Moreira et al., 2018).

### 2.10 Statistical analysis

All numeric data were derived from three independent experiments and are presented as mean ± standard deviation. We used GraphPad Prism (GraphPad Software Inc.) and ImageJ software for analyses. The statistical analyses were done by one-way analysis of variance (ANOVA) followed by Dunn’s or Dunnet’s tests. Statistical significance is denoted by asterisks: *p ≤ 0.05, **p ≤ 0.01, ***p ≤ 0.001, and ****p ≤ 0.0001.

## 3 Results

### 3.1 SRPK1/2 expression in patients with melanoma

**FIGURE 1:**
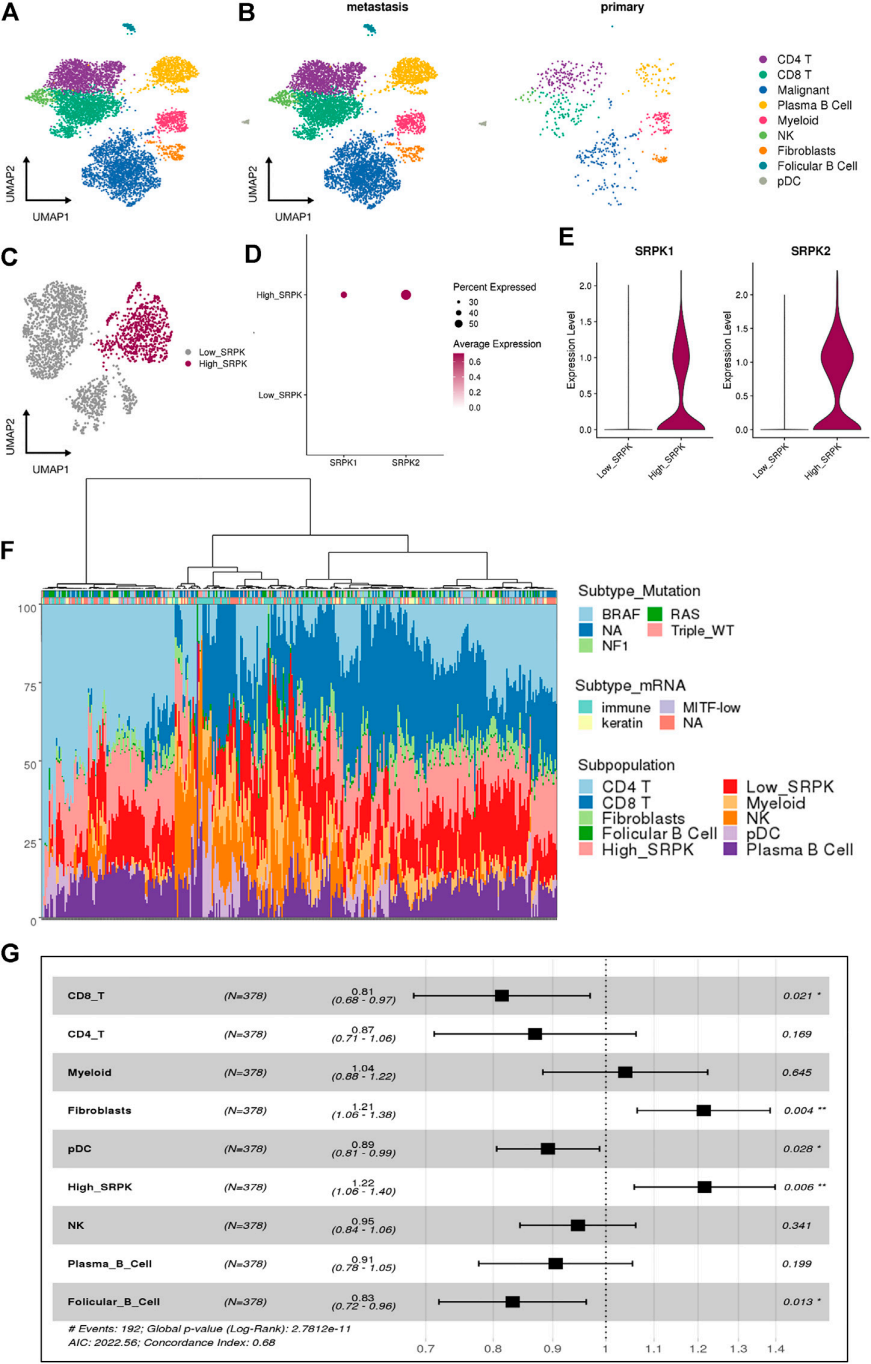
Single-cell RNA sequencing reveals a relationship between high SRPK1/2 expression in malignant cells and poor clinical outcomes in melanoma patients. **(A)** Analysis of 6,696 single cells from melanoma samples. Each cell is depicted in the UMAP and color-coded for broad cell types. **(B)** UMAP and color-coded for broad cell types and split by tumor sites. **(C)** Clusters of malignant cells showing the segregation by High_ and Low_SRPK malignant cells represented in the UMAP. **(D)** Dot plot and **(E)** violin plot showing the expression of SRPK1 and SRPK2 among malignant High_SRPK and Low_SRPK cells. **(F)** Bar plot of the estimated cellular composition in the melanoma TME from the TCGA patient cohort. **(G)** Forest plot representing the survival hazard ratio of relative fractions, divided into quartiles, for each cell type in a multivariate analysis (*p* < 0.001, HR = 1.4, 95% CI = 1.2–1.6).

scRNA-Seq allows accessing the complexity of the transcriptome of individual cells in a TME (Tang et al., 2009; Hwang et al., 2018). To investigate the expression of SRPK1 and SRPK2 in human melanoma at single-cell resolution, we analyzed a dataset from human melanoma biopsies containing 6,696 cells identified by canonical lineage markers (Figure 1A). Most cells analyzed were derived from metastatic samples while only 592 cells (8.8%) were derived from primary tumors (Figure 1B).

We investigated malignant cells for CNV signals, by comparing them with other immune and stromal cells from the melanoma TME (Supplementary Figure S1A, B), to confirm that most melanocytes were indeed malignant. Of 2,147 malignant cells, 97% were classified as “High_CNV” and the remaining ones returned a lower score than the cutoff value (Supplementary Figure S1C, D).

Using the previous classification according to SRPK1/2 expression (Moreira et al., 2022), we grouped the clusters of malignant cells that had low expression of SRPK1/2 (0, 2, 3, and 4) in relation to the cluster that had a high percentage of cells expressing SRPK1/2 (1) (Figure 1C, Supplementary Figure S1E,F). We named these clusters as “Low_SRPK” (1,485 cells) and “High_SRPK” (662 cells), respectively. When we analyzed the “High_SRPK” cluster in more detail, we found higher expression of SRPK2 compared with SRPK1 (Figures 1D,E).

To investigate the clinical outcomes of patients whose melanoma tumors bear “High_SRPK” malignant cell subpopulations, we performed a deconvolution analysis using cell signatures from scRNA-Seq to estimate the proportion of these cells in the bulk RNA-Seq samples (Figure 1F). At least three groups of melanoma samples could be clustered regarding their TME profile estimates: one with a great proportion of CD4^+^ T cells and “High_SRPK” malignant cells; a second group with a great proportion of myeloid and natural killer (NK) cells, and a high proportion of “Low_SRPK” malignant cells; and a third group with a high proportion of “High_SRPK” malignant cells and CD8^+^ T cells. Then, we calculated the HR of the relative fractions of each cell type population using survival time data, first in a univariate model and later in a multivariate analysis (Supplementary Table S1). There was an elevated HR (poorer prognosis) related to High_SRPK malignant cells as an independent variable in the multivariate model (*p* < 0.006, HR = 1.22, 95% CI = 1.06–1.4), indicating that high SRPK1/2 expression in malignant cells is associated with an unfavorable prognosis (Figure 1G). We also observed a significant poor prognosis related to fibroblasts (*p* < 0.04, HR = 1.21, 95% CI 1.06–1.38) and a lower HR related to CD8 T cells (*p* < 0.021, HR = 0.81, 95% CI 0.68–0.97), plasmocitoid dendritic cells (pDC) (*p* < 0.028, HR = 0.89, 95% CI 0.81–.0.99), and follicular B cells (*p* < 0.13, HR = 0.83, 95% CI 0.72–0.96).

### 3.2 Effect of genomic modification on cell proliferation, invasion, and colony formation

Tumor development is a multistep process that occurs from genetic mutations leading to abnormal cell proliferation. Subsequently, the tumor progresses by acquiring properties such as cell survival, local invasion, intravasation into blood vessels, extravasation, immune evasion, and colonization of the target tissue to form metastasis (Fares et al., 2020).

**FIGURE 2:**
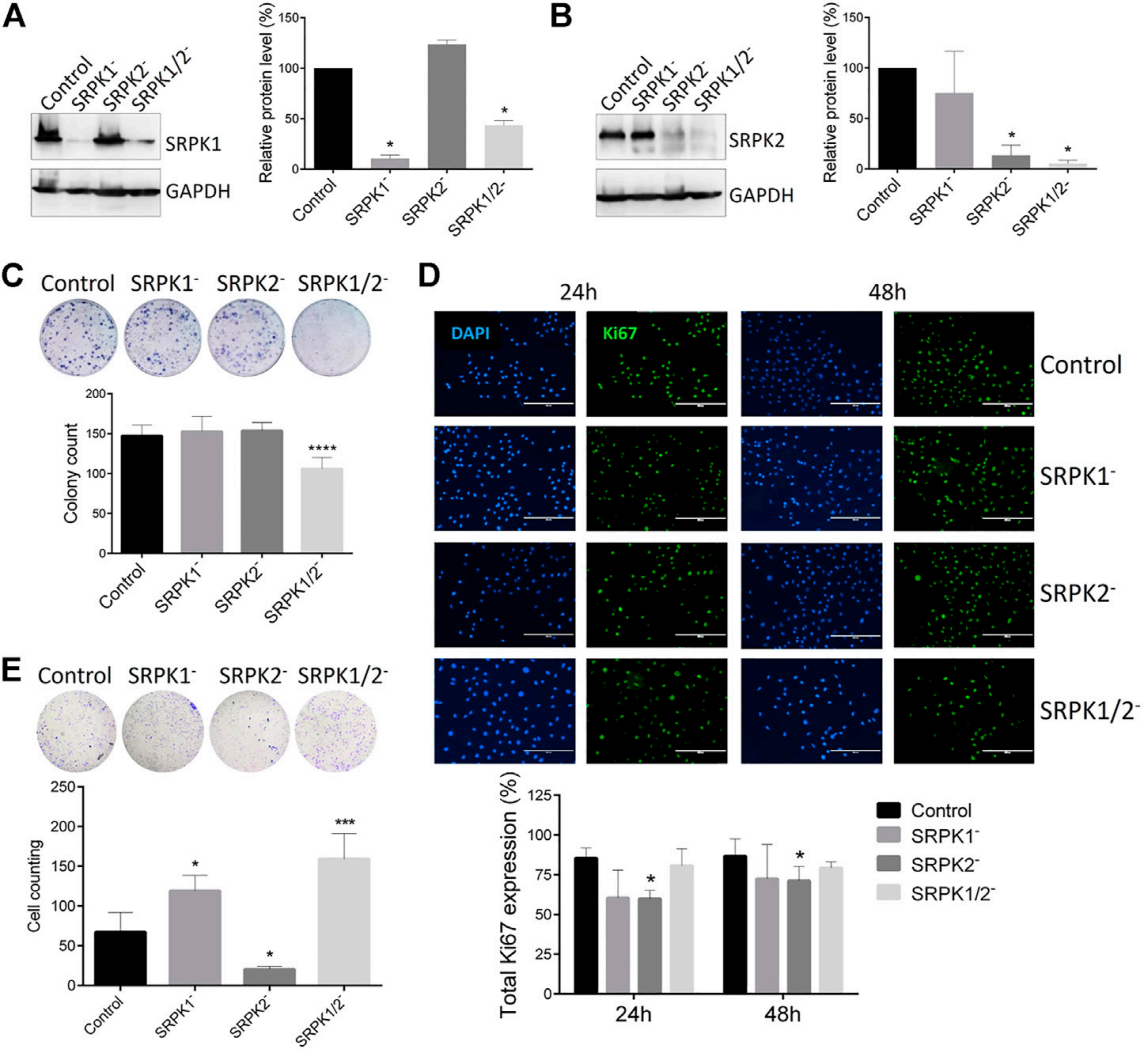
Effect of genetic targeting of SRPK1, SRPK2, or both in B16F10 cells. **(A,B)** Western blot analysis of B16F10 lineages generated by CRISPR-Cas9 genome edition. “Control” denotes cells transduced with the empty LentiCRISPRv2 vector. The SRPK1 or SRPK2 protein levels were normalized to GAPDH. The bars represent the mean ± standard deviation (SD) from duplicate experiments. **(C)** The clonogenic assay was performed by toluidine blue staining after 14 days. Simultaneous genetic targeting SRPK1 and SRPK2 impairs colony formation in B16F10 cells. **(D)** The effect of SRPK genetic targeting in cell proliferation *in vitro*. The cells were fixed at 24 and 48 h and stained for Ki67, an endogenous proliferation marker. Representative images of staining are shown. The results are expressed as proliferative cells in relation to the total number of cells in each field (represented by the nucleus stained with DAPI). Values in the bar graphs represent the mean ± SD (n ≥ 5 fields). Scale bar 200 μm. **(E)** SRPK2 genetic targeting strongly decreases B16F10 invasion of B16F10 cells, while depletion of SRPK1 or simultaneous SRPK1/2 increases the invasiveness. The graph shows the quantitative analysis of the Matrigel chamber invasion. The data are expressed as the mean ± SD from triplicate experiments. *p ≤ 0.05, ***p ≤ 0.001, ****p ≤ 0.0001 versus control by Dunnett’s test.

Considering the clinical correlations obtained from single-cell analysis, we approached a well-known murine metastatic melanoma model to better investigate the potential role of SRPK1/2 *in vitro* and *in vivo*. In a first set of experiments, we obtained the B16F10 cell genome edited for SRPK1, SRPK2, or both loci (Figures 2A,B). We then evaluated the effect of SRPK1/2 genetic targeting on processes related to the metastatic capacity of B16F10 cells. We found that only simultaneous SRPK1/2 genetic targeting was able to impair the colony formation capacity *in vitro* (Figure 2C). On the other hand, SRPK2 genetic targeting could decrease cell proliferation and the Matrigel matrix invasion capacity, while SRPK1 or simultaneous SRPK1/2 genetic targeting did not significantly affect the cell proliferation and led to an increased invasion capacity (Figures 2D,E).

### 3.3 Effect on F-actin polymerization

**FIGURE 3:**
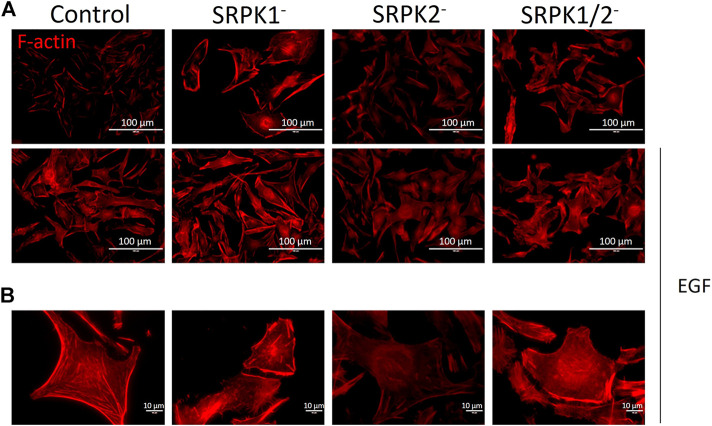
SRPK2 genetic targeting affects actin polymerization in B16F10 cells. **(A)** Cells were treated with EGF and then fixed. F-actin was detected by fluorescence microscopy using Rhodamine Phalloidin. Scale bar, 100 µm. **(B)** Higher magnification images showing the low fluorescence intensity in B16F10 SRPK2-targeted cells, indicating the lowest amount of F-actin in these cells. The scale bar 10 µm.

Impaired function of the actin cytoskeleton can be related to all stages of metastasis (Fife et al., 2014). We evaluated whether SRPK1/2 genetic targeting could affect the actin cytoskeleton. As reported previously (Rijken et al., 1991), EGF treatment stimulated actin polymerization to the F-actin form in B16F10 control cells (Figures 3A,B). Independently of EGF stimulus, there was lower fluorescence intensity in SRPK2-modified cells, indicating that the SRPK2 genetic targeting impaired the formation of F-actin (Figures 3A,B). In SRPK1- or SRPK1/2-targeted cells, there was no difference in the fluorescence intensity (Figures 3A,B).

### 3.4 Effect on melanoma progression *in vivo*

**FIGURE 4:**
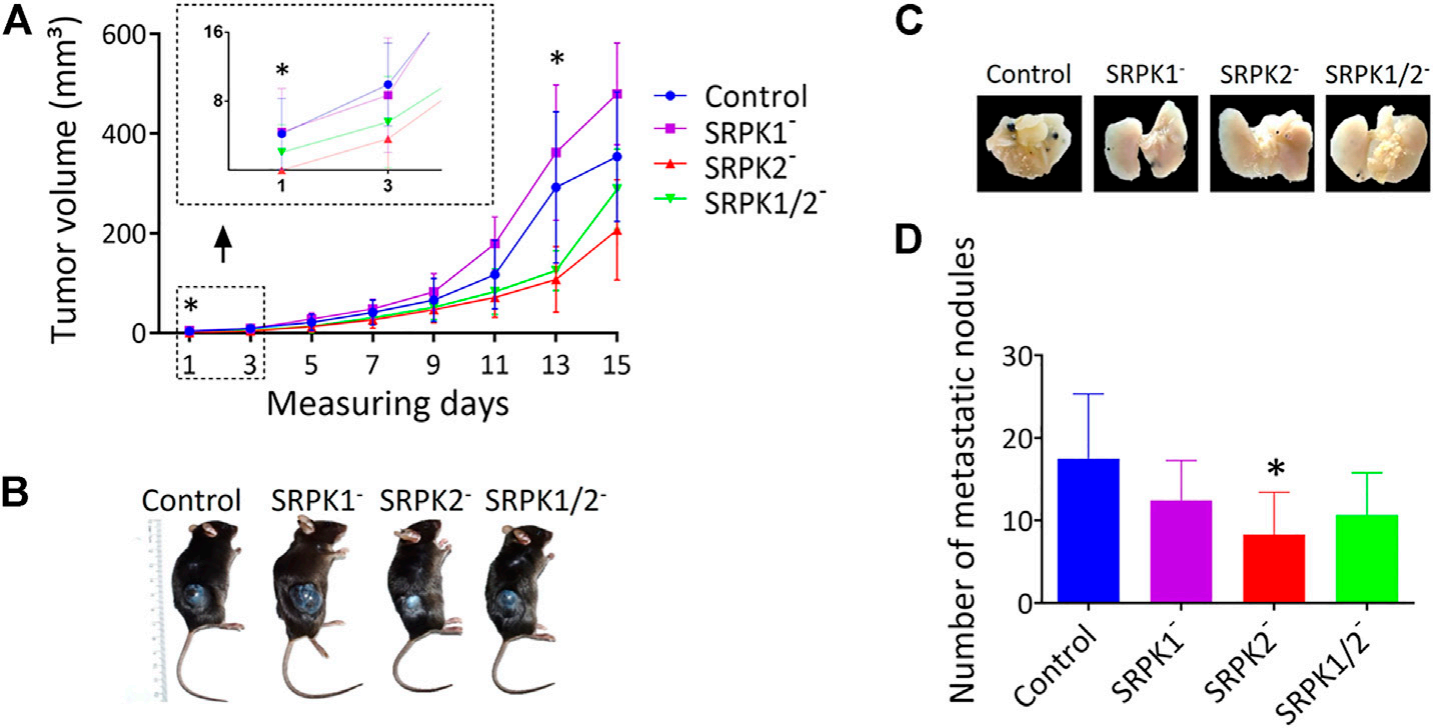
The effects of SRPK genetic targeting on tumor growth *in vivo*. **(A)** Genetic targeting of SRPK2 impairs subcutaneous tumor development in mice. The animals were observed daily until the tumor was identified in the first animals (day 1). The volume of subcutaneous tumors was measured every 2 days for 15 days **(B)** Representative photographs of tumor formation 15 days after inoculation with B16F10 cells. **(C)** SRPK2 genetic targeting impairs lung nodules development in mice. Representative images of pulmonary metastatic foci produced 21 days after intravenous injection of B16F10 cells. **(D)** Lungs were fixed in 4% (v/v) paraformaldehyde solution and the number of metastatic nodules on the surface was quantified. Most of the counted nodules were only visualized with the aid of a magnifying glass due to their small size. The graph shows the number of pulmonary metastatic foci. All results are expressed as the mean ± SD. One-way ANOVA followed by Dunn’s test was performed (*p < 0.05).

We evaluated the effect of SRPK1/2 genetic targeting on subcutaneous melanoma growth and development in mice. First, we identified the onset of tumors in animals inoculated with control and SRPK1- and SRPK1/2-targeted cells, but not in animals inoculated with SRPK2-targeted cells (Figure 4A). Although the experimental groups did not differ at the end point, the tumor volumes of animals inoculated with modified SRPK2 cells remained lower on day 13 of the experiment (Figure 4A). This result suggests that SRPK2 targeting impaired tumor development. Representative images of the experimental animals are shown in Figure 4B.

In another set of experiments, we investigated whether the genetic modifications performed could affect tumor induction through the caudal vein. Consistent with the data obtained subcutaneously above, SRPK2 genetic targeting significantly reduced the formation of nodules in lungs while SRPK1 or SRPK1/2 targeting had no significant effect (Figure 4D). The representative images in Figure 4C show the lungs of the animals containing the nodules.

## 4 Discussion

Several SRPK1/2-related roles in cancer have been reported, such as angiogenesis (Gammons et al., 2014), proliferation (Jang et al., 2008), cell migration and EMT (Wang et al., 2017), metastasis (van Roosmalen et al., 2015; Zhuo et al., 2018), lipid biosynthesis (Lee et al., 2017), genome stability maintenance (Sridhara et al., 2017), and drug resistance mediation (Wang et al., 2019). We previously demonstrated the anti-metastatic effect of pharmacological inhibitors targeting the SRPK1/2 family in melanoma (Gammons et al., 2014; Moreira et al., 2018; Moreira et al., 2022). As selective SRPK pharmacological inhibitors are still unknown, the role of SRPK1 or SRPK2 activity in melanoma remains obscure. So, our main objective in this work was to evaluate the possible differential roles of SRPK1 and SRPK2 in melanoma.

Malignant melanoma is highly heterogeneous in terms of cellular and molecular composition, presenting a complex TME characterized by intense infiltration of various immune cell subsets (Jorge et al., 2020; Liu et al., 2021). This complex network of cells and molecules communicates intimately and affects prognosis by inducing tumor resistance to therapeutic interventions (Zeng et al., 2021). By using scRNA-Seq data, we verified that SRPK1/2 overexpression in malignant melanoma cells is linked to poor clinical outcomes. Interestingly, this cluster of malignant melanoma cells exhibited higher levels of SRPK2 than SRPK1. This finding supports the evidence that the increased malignant potential of melanoma cells, along with the consequent worsening in patient prognosis, should also be related to SRPK2 activity.

Although the best-known function of SRPKs is classically related to regulation of pre-mRNA splicing (Wang et al., 1998), new findings have shown that these kinases can act broadly, depending on their expression levels and subcellular localization, the signaling pathways in which they are involved, and according to the cellular state (Nikolakaki et al., 2022). Here, we showed that genetic targeting of SRPK2 affected actin filament polymerization by decreasing the formation of F-actin in B16F10 cells. In addition, genetic targeting of SRPK2 impaired cell proliferation and invasion, a process related to the ability of B16F10 cells to metastasize. These results are closely related to each other, as actin cytoskeleton remodeling is fundamental during all stages of metastasis. For instance, cell migration is facilitated by the formation of protrusions of specialized membranes such as invadopodia, lamellipodia, and filopodia, which are dependent of the actin filament polymerization to disrupt basement membranes, and to invade tissues, blood, and lymph vessels (Fife et al., 2014). The details of how SRPK2 interferes with the dynamics of actin cytoskeleton remodeling remain to be clarified. Hypothetically, SRPK2 could act directly on actin or indirectly through phosphorylation and regulation of actin-binding proteins. This work paves the way for future investigations into the role of SRPK2 in this process.

On the other hand, SRPK1 genetic targeting did not significantly impair cell proliferation, increased invasion activity, and seemed to apparently increase tumor progression *in vivo*. Although we cannot affirm that SRPK1 should promote melanoma progression (the differences were not significant), this hypothesis could not be discarded and should be better evaluated elsewhere. Indeed, SRPK1 has been shown to be necessary for the recruitment of PHLPP1, a phosphatase that dephosphorylates and thus inactivates AKT. When SRPK1 is misregulated, PHLPP1 is not recruited efficiently and AKT remains phosphorylated and active, promoting tumorigenesis (Wang et al., 2014). So, different studies have reported that depending on the biochemical context, SRPK1 overexpression (Hayes et al., 2007; Odunsi et al., 2012; Siqueira et al., 2015; van Roosmalen et al., 2015) or downregulation (Schenk et al., 2004; Hayes et al., 2006; Wang et al., 2014) have pro-tumoral effects.

In summary, we have shown that in metastatic melanoma, high expression of SRPK1/2, especially SRPK2, contributes to the poor prognosis of patients. In addition, SRPK2 genetic targeting disrupted actin filament formation and decreased the ability of B16F10 cells to proliferate and invade. Consistently, genetic targeting of SRPK2, but not SRPK1, impaired the formation of pulmonary nodules and the development of subcutaneous melanoma in mice. Finally, the more prominent impact of SRPK2 genetic targeting in impairing the development of murine melanoma in comparison to SRPK1 suggests that selective SRPK2 inhibition may be considered in further drug discovery efforts.

## Data Availability

Publicly available datasets were analyzed in this study. The clinical and bulk RNA sequence data can be found in the TCGA database (https://portal.gdc.cancer.gov), using the identifiers “Skin Cutaneous Melanoma” or “SKCM”. The scRNA-Seq data and its associated metadata can be found in the GEO Database under accession number GSE115978. The previously scripts developed for the analyzed malignant cells are available at the GitHub repository “SRPK at single-cell resolution” (https:// github.com/bioinformatics-inca/srpk_single-cell) (Rapozo and Santos, 2022a), and the script developed for this study are available at GitHub repository “Role of SRPK in melanoma prognosis” (https://github.com/bioinformatics-inca/clinical_impact_SRPK) (Rapozo and Santos, 2022b).

## Abbreviations

SRPK, serine/arginine protein kinase; CRISPR-Cas9, clustered regularly interspaced short palindromic repeat (CRISPR)-associated protein 9; EMT, epithelial-mesenchymal transition; VEGF, vascular endothelial growth factor; EGF, epidermal growth factor; EGFR, epidermal growth factor receptor; PI3K, phosphoinositide 3-kinase; AKT, protein kinase B; SRSF1, serine and arginine rich splicing factor 1; ERK, mitogen-activated protein kinase; mTORC1, rapamycin complex 1; Rac1, Ras-related C3 botulinum toxin substrate 1; scRNA-seq, single-cell transcriptome sequencing; TME, tumor microenvironment; CNV, copy number variation; TCGA, cancer genome atlas; HR, hazard ratio; CI, confidence interval; GAPDH, glyceraldehyde-3-phosphate dehydrogenase; HRP, horseradish peroxidase; sgRNA, single guide RNA sequence; MOI, multiplicity of infection, FBS, fetal bovine serum; UMAP, uniform manifold approximation and projection; PHLPP1, PH domain leucine-rich repeat-containing protein phosphatase 1.
