# Supplementary material for: Impaired expression of serine/arginine protein kinase 2 (SRPK2) affects melanoma progression
Source: Front Genet. 2022 Sep 23;13:979735. doi: 10.3389/fgene.2022.979735 (PMC9537589; doi:10.3389/fgene.2022.979735)
Supplement: Supplementary file 1 [file Table1.PDF]

**Supp. Table 1.** Univariate analysis of cell types in the melanoma tumor microenvironment (p-value <0.05 represented as *).

| Celltype | beta | HR (95% CI for HR) | wald.test | p.value |
|---|---|---|---|---|
| CD4_T | 0.11 | 1.1 (1-1.2) | 3.8 | 53 |
| CD8_T | -0.13 | 0.88 (0.8-0.97) | 6.2 | 0.013* |
| Fibroblasts | 0.29 | 1.3 (1.2-1.5) | 30 | 3.4e-08* |
| Folicular_B_Cell | -0.26 | 0.77 (0.69-0.87) | 19 | 1.3e-05* |
| High_SRPK | 0.29 | 1.3 (1.2-1.5) | 27 | 1.8e-07* |
| Low_SRPK | 37 | 1 (0.93-1.2) | 0.41 | 0.52 |
| Myeloid | -0.16 | 0.85 (0.77-0.94) | 9.3 | 0.0022* |
| NK | -98 | 0.91 (0.84-0.98) | 5.8 | 0.016* |
| pDC | -0.22 | 0.8 (0.74-0.87) | 26 | 2.8e-07* |
| Plasma_B_Cell | 0.17 | 1.2 (1.1-1.3) | 8.2 | 0.0041* |
