# Supplementary material for: Impaired expression of serine/arginine protein kinase 2 (SRPK2) affects melanoma progression
Source: Front Genet. 2022 Sep 23;13:979735. doi: 10.3389/fgene.2022.979735 (PMC9537589; doi:10.3389/fgene.2022.979735)
Supplement: Supplementary file 2 [file Image1.pdf]

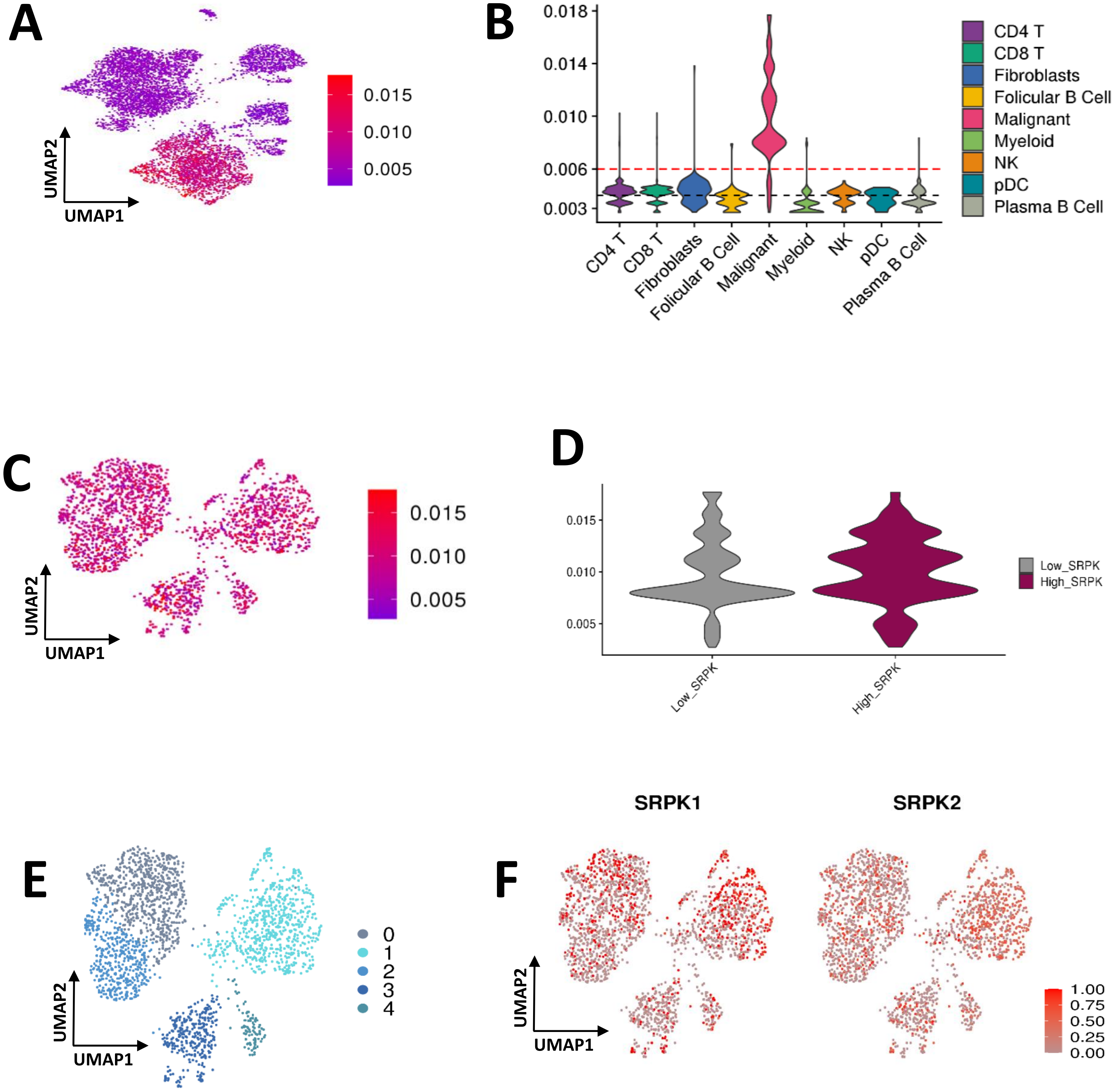

Supplementary Figure 1. **Copy number variation (CNV) across single-cell RNA-Seq melanoma data. (A)** UMAP depicting all broad cell type clusters; the color scale shows the cnv scores. **(B)** Violin plot showing the cnv score for the different cell types. The red line shows the cutoff, and the black line shows the average of the cnv score in the other cells in the tumor microenvironment. **(C)** UMAP showing the cnv score scale for the malignant cells subset. **(D)** Violin plot showing the cnv score for the "Low_SRPK" and "High_SRPK" subpopulations of malignant cells. **(E)** UMAP showing the original label clusters, and **(F)** the expression of SRPK1/2.
